# Using a biopsychosocial approach to examine differences in post-traumatic stress symptoms between Arab and Jewish Israeli mothers following a child’s traumatic medical event

**DOI:** 10.1186/s12939-021-01429-y

**Published:** 2021-03-31

**Authors:** Sewar Hussein, Yaara Sadeh, Rachel Dekel, Efrat Shadmi, Amichai Brezner, Jana Landa, Tamar Silberg

**Affiliations:** 1 Department of Psychology, Bar-Ilan University, Ramat-Gan, Israel; 2 The Louis and Gabi Weisfeld School of Social Work, Bar-Ilan University, Ramat-Gan, Israel; 3 Department of Pediatric Rehabilitation, The Edmond and Lily Safra Children’s Hospital, Sheba Medical Center, Ramat-Gan, Israel; 4 The Cheryl Spencer Department of Nursing, Faculty of Social Welfare & Health Sciences, University of Haifa, Haifa, Israel; 5 Sackler Faculty of Medicine, Tel Aviv University, Tel Aviv, Israel

**Keywords:** Trauma, Pediatric, Rehabilitation, Ethnic, Mental-health

## Abstract

**Background:**

Parents of children following traumatic medical events (TMEs) are known to be at high risk for developing severe post-traumatic stress symptoms (PTSS). Findings on the negative impact of TMEs on parents’ PTSS have been described in different cultures and societies. Parents from ethnic minority groups may be at particularly increased risk for PTSS following their child’s TME due to a host of sociocultural characteristics. Yet, differences in PTSS manifestation between ethnic groups following a child’s TME has rarely been studied.

**Objectives:**

We aimed to examine: (1) differences in PTSS between Israeli-Arab and Israeli-Jewish mothers, following a child’s TME, and (2) risk and protective factors affecting mother’s PTSS from a biopsychosocial approach.

**Methods:**

Data were collected from medical files of children following TMEs, hospitalized in a Department of Pediatric Rehabilitation, between 2008 and 2018. The sample included 47 Israeli-Arab mothers and 47 matched Israeli-Jewish mothers. Mothers completed the psychosocial assessment tool (PAT) and the post-traumatic diagnostic scale (PDS).

**Results:**

Arab mothers perceived having more social support than their Jewish counterparts yet reported higher levels of PTSS compared to the Jewish mothers. Our prediction model indicated that Arab ethnicity and pre-trauma family problems predicted higher levels of PTSS among mothers of children following TMEs.

**Conclusions:**

Despite reporting higher social support, Arab mothers reported higher levels of PTSS, as compared to the Jewish mothers. Focusing on ethnic and cultural differences in the effects of a child’s TME may help improve our understanding of the mental-health needs of mothers from different minority groups and aid in developing appropriate health services and targeted interventions for this population.

## Introduction

The National Child Traumatic Stress Network defines pediatric medical traumatic stress as “a set of psychological and physiological responses of children and their families to pain, injury, serious illness, medical procedures, and invasive or frightening treatment experiences”. These responses frequently involve (but are not limited to) post-traumatic stress symptoms (PTSS), including re-experiencing, avoiding reminders of the trauma, and hyperarousal among children and their families. These patterns of symptoms prove to be cross-cultural, with multiple studies emphasizing the impact of a child’s medical condition on parents’ level of distress in various societies and ethnic groups worldwide [1–4].

Parents of children who have experienced a traumatic medical event (TME) are known to be at a high risk for developing various degrees of stress responses [5–8]. Recently, the integrative biopsychosocial model of PTSS was developed by Marsac and colleagues [9, 10], with the aim of identifying child, family and environmental factors associated with children’s and parents’ risk for developing PTSS. ﻿These authors incorporated biological, psychological, social, and environmental risk factors throughout the recovery from a traumatic event. According to the model, each of the factors can be present across different time points: before the trauma (i.e., pre-trauma), during the acute phase following the trauma (i.e., peri-trauma), or during the more chronic phase (i.e., post-trauma). ﻿In this model, psychosocial factors are presented as being important predisposing factors for parents’ acute stress responses, with the severity of early distress responses proposed as predictors of long-term PTSS.

The current study wished to examine a model in which﻿ biological (i.e., age, gender and injury etiology), psychological (i.e., child’s and siblings’ emotional and behavioral problems), and environmental (i.e., family functioning, community support, ethnicity) pre-trauma factors contribute to mothers’ PTSS following a child’s TME. Specifically, we aimed to understand differences in PTSS between Arab and Jewish Israeli mothers of children following TMEs, within a multicultural society. Improved understanding of the relationship between risk and protective factors and PTSS, while taking into account the role of ethnic and socio-cultural factors, may improve understanding and management of pediatric medical traumatic stress.

Research showed that a child’s pre-trauma biological factors, such as gender and age, are associated with parents’ PTSS, with female gender and younger age as risk factors for parental PTSS following various types of traumatic events [11–13]. In addition, characteristics of a child’s medical condition (e.g., type of tumor [7], size of burn [14], child’s functional status [15]) have been positively associated with intensity of parents’ traumatic response, as well as with parent’s general psychological distress [12, 16–18]. However, the role of injury severity in predicting parental PTSS is still unclear, with some studies indicating a positive relationship between injury severity and parents PTSS [19] and others failing to show such association [16]. A child’s pre-existing emotional and behavioral problems, as well diagnosis of PTSD following a TME, were also shown to be associated with a higher risk for parental PTSD [6, 7].

In addition to the child’s personal factors, family-related factors were also shown to be associated with parents’ post-traumatic responses. For example, younger parental age has been associated with higher levels of PTSS [7, 12, 15]. Higher parental education levels were linked with positive coping strategies and lower levels of maternal distress [20]. In contrast, other studies have indicated that more educated mothers of children with chronic medical conditions experienced higher levels of anxiety and psychological distress than less educated mothers [16]. Psychosocial factors, such as parents’ anxiety or depression have also been associated with parents’ emotional distress following a child’s TME, accounting for approximately 37% of overall variance in their distress responses [7]. These factors have also been associated with parents’ risk for developing PTSD [4].

Environmental and social factors may also play a significant role in the manifestation of PTSS. In general, findings on the negative impact of a TME on parents’ emotional state have been described in different cultures and societies worldwide [1, 4, 21], even though the literature indicates a difference in the frequency of PTSS and/or PTSD, and even in referral to mental health treatments, among different ethnic groups [22, 23]. It was, however, argued that the belonging to a minority ethnic group may contribute to increased vulnerability [24, 25] to PTSS, thus resulting in an increased risk for parental traumatic stress responses following a child’s TME.

Ethnic differences in parental PTSS and/or PTSD might have various sources. A recent review by Asnaani and Hall-Clark [26] addressed the role of cultural factors, such as norms related to symptom disclosure, reporting style, cultural interpretations of symptoms and distress, and of specific coping styles, all associated with ethnical differences in PTSD risk. Nevertheless, with regard to identifying and treating PTSD, research has repeatedly shown that minority groups encounter disparities in access to social and economic resources (health care, education, etc.) [27]. Members of minority groups may also have fewer resources; thus, any loss of a single resource may negatively affect their ability to recover from a medical traumatic event [28, 29]. Moreover, the perception of discrimination within minority groups may itself prevent individuals from seeking social support and/or making use of available resources after traumatic events [27, 30]. As suggested by the biopsychosocial model, social support is considered an important environmental factor associated with reduced parental distress following a child’s TME [9, 10]. Furthermore, because culture and attribution to a specific ethnic group may influence people’s beliefs, attitudes, expectations and behavior regarding the role of social support, the latter should be acknowledged when treating parents and families following TMEs [31, 32].

In Israel, a multicultural pluralistic society that includes diverse ethnic and religious identities, the Arab population constitutes a relatively large minority group (20.3% of the general population, according to the Israel Central Bureau of Statistics [33]). Arabs in Israel are at a significant disadvantage for a wide range of health indicators compared with the Jewish majority, including shorter life expectancy and higher infant mortality rates [34]. Traumatic injuries in children are, unfortunately, no exception. According to a national report on child injuries in Israel [35], the relative risk of hospitalization, for an Arab child is 2.5 times greater than the risk for a Jewish child. Moreover, as the severity of injury increases, the proportion of Arab children among those hospitalized increases as well. Additionally, Arab children have different injury patterns, resulting in a tendency for them to be injured more frequently in the types of accidents that have more severe consequences (motor vehicle accidents, burns, falls, etc. [35]. According to the report, despite universal health coverage under the Israeli Health Insurance Law, the higher risks among Arab children are related to differential access to high-quality preventive medical care. Subsequently, Arab children and their families might be at higher risk of developing post-traumatic responses, which, in turn, may also affect their recovery.

Differences in traumatic stress responses between Israeli Arab and Jewish ethnic groups were reported [28, 36, 37] indicating that Arabs are at increasingly significant risk for the development of PTSS due to various traumatic events such as terrorism and political violence. This finding was also evident among Jewish and Arab Israeli youth, with the latter reporting more severe post traumatic symptoms [38]. Similarly, a study conducted among children in Israel showed that Arab children reported higher levels of PTSD than their Jewish counterparts [37]. Such associations, however, were not examined in relation to a child’s TME.

**Fig. 1:**
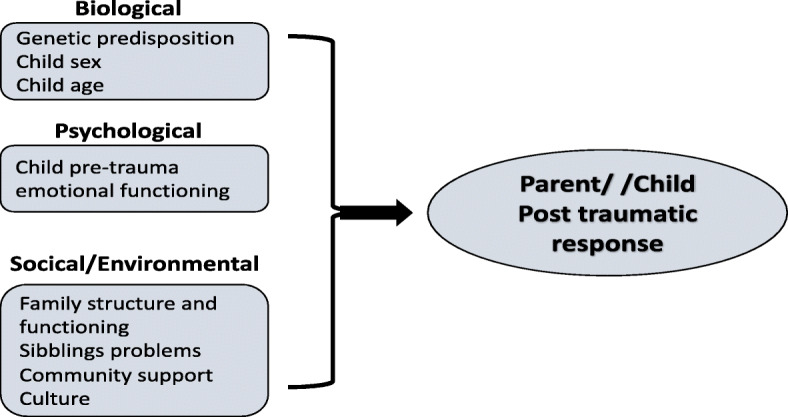
A bio-psycho-social model for the prediction of post traumatic responses following a child’s TME (adapted from Marsac et al., 2014)

Research on parental response to children with special needs in Arab populations in Israel is limited. One study of Arab mothers of children with special needs in Israel found that they had higher overall stress levels than Arab mothers of healthy children. Moreover, the increase in stress levels was associated with these mothers’ reporting of poorer well-being [39]. However, the levels of symptomatology among the Arab mothers were not compared with those of Israeli Jewish mothers, limiting the ethnicity-related conclusions that could be drawn from the study. Moreover, no previous study has addressed the role of the different pre-trauma factors associated with mothers’ PTSS within the biopsychosocial framework (Fig. 1), in the context of specific ethnic minority groups (such as that of the Israeli-Arab population). Thus, an in-depth examination of post-traumatic responses among mothers of children following TMEs within the Israeli Arab and Jewish population is warranted.

The major aim of this study was to examine differences between Israeli Arab and Jewish mothers’ post-traumatic responses following their child’s TME. Furthermore, our emphasis was on analyzing how well the biopsychosocial [9, 10] framework explains mother’s traumatic stress responses, specifically how the different components of the model explain the differences in mother’s traumatic stress responses. We hypothesized that Israeli-Arab mothers will self-report higher levels of PTSS than will their Jewish counterparts and that the levels of high PTSS will be associated with child, family and social pre-trauma risk factors.

## Methods

### Setting

The current study was part of a larger study conducted in the Department of Pediatric Rehabilitation at the Sheba Medical Center, Israel, assessing long-term trajectories of PTSS following a child’s TME. As part of the admission process in the department, each family was asked by their psychologist / social worker to complete a psychosocial protocol (composed of questionnaires for the children and their parents) in order to screen for psychosocial risk and for mother’s PTSS. The current study employed a retrospective design of archival clinical and questionnaire data of children hospitalized during the period between 2008 and 2018. All study procedures were approved by the Institutional Review Board at the Sheba Medical Center.

### Participants

Participants were eligible for inclusion in the analysis if they (1) had experienced a TME within the 3-month period prior to their hospitalization in the rehabilitation department, (2) agreed to complete the questionnaires during their admission to the department, and (3) had sufficient Hebrew-language proficiency to complete the assessment questionnaires. Potential participants were excluded from analysis if (1) their medical condition or cognitive limitations prevented completion of the clinical data, (2) their injuries were due to family violence or suspected child abuse, or (3) the child or parent was subject to legal proceedings related to the injury, the child or parent was the perpetrator of violence related to the injury, or the child or parent declined to complete the questionnaires. ﻿.

A total of *N* = 62 Israeli-Arab families were admitted to the pediatric department following a child’s TME between 2008 and 2018, with *N* = 7 without sufficient Hebrew-language proficiency to complete the assessment questionnaires; *N* = 5 child’s TME occurred more than 3-months from hospitalization at the pediatric department; and *N* = 3 did not agree to complete the questionnaires during admission to the department. Subsequently, a sample of *N* = 47 Israeli-Arab mothers was matched with a sample of *N* = 47 Israeli-Jewish mothers (out of a total of *N* = 326 Israeli-Jewish mothers), based on child’s gender, type of TME and age at the time of TME. Matching was done manually by two of the authors (H.S and T.S) according to an index created to consider the matching criteria of (1) sex, (2) age and (3) TME etiology. In cases in which more than one case in the Jewish sample qualified for all matching criteria (mainly for the ABI and orthopedic groups) a random software (https://www.randomizer.org) was used to select a single case. Furthermore, any conflicts were resolved via discussion. No significant differences were found between the mothers groups for gender (*χ*^2^_(1)_ = 0.49, *p* = 0.82) or age at trauma (t_(92)_ = − 0.48, *p* = 0.63) (see Tables 1 and 2 for sample characteristics).

**Table 1:** Distribution of the children in the sample in the two ethnic groups according to age at trauma and gender

Ethnicity	Gender (N)	Mean age at TME, yrs (SD)
**Jewish (*N*** **= 47)**	Male (33)	9.23 (5.49)
	Female (14)	9.36 (5.91)
**Arab (*N*** **= 47)**	Male (34)	9.62 (6.10)
	Female (13)	9.77 (5.98)

*TME* traumatic medical event

**Table 2:** Distribution of the different TME etiologies among the children in the sample according to the different ethnic groups

Injury type	Jewish Frequency (%)	Arab Frequency (%)
Acquired brain injury (ABI)	24 (51)	22 (46.8)
Spinal cord injury (SCI)	3 (6.4)	5 (10.6)
Cerebral Palsy (CP) Operation	2 (4.3)	1 (2.1)
Orthopedic Injury	11 (23.4)	10 (21.3)
Multi-trauma	7 (14.8)	8 (18.9)
**Total**	**47 (100)**	**47 (100)**

*TME* traumatic medical event

Type of TME in the sample was sorted into six groups, with 56.3% of children diagnosed with ABI (acquired brain injury), 21.9% with orthopedic injuries, 8.3% with SCI (spinal cord injury), 4.2% having had an orthopedic operation due to CP (cerebral palsy) and 9.3% having had a multi-trauma injury. The etiologies of the TMEs were equally distributed between the two ethnic groups (χ^2^_(5)_ = 1.86, *p* = 0.87) (see Table 2).

The dependent variable in our study was mothers’ levels of PTSS. Predictors were mother’s ethnicity (Arab vs. Jewish), child-related factors (age and gender) and family’s pre-trauma risk factors (family structure and resources, family problems, child problems, sibling problems and family social support).

Sample size analysis using G*power3.1 was conducted to examine a prediction model using *Test family F-test, with the specific statistical test of multiple regression analysis: Fixed model, R*^*2*^ *increas*e for levels of PTSS among mothers of children following TMEs. With the nine predictions required for the regression analysis, the sample size needed for a medium effect (d = 0.2), a power of 0.8 and significance level of *p* < 0.05 was 88 participants.

### Measures

#### Predictors

##### Ethnicity

Indication of ethnicity (Arab or Jewish) was extracted from background information provided by families upon admission to the department.

##### Child-related factors

Child’s age, gender and type of TME were extracted from medical records.

##### Psychosocial risk factors

The Psychosocial Assessment Tool (PAT [40]) was used to screen for psychosocial risk in the context of the family. The questionnaire has been used among the pediatric population, with children across a broad age range, from infants to adolescents [41]. The PAT evaluates specific areas of psychosocial risk organized in seven subscales: family structure and resources (8 items; e.g., areas of financial difficulties), social support (4 items; e.g., provision of emotional/financial support), sibling problems (16 items; e.g., have school or learning difficulties), child problems (16 items; e.g., level of distraction), family problems (15 items; e.g., marital difficulties, separation), parent stress reactions (3 items; e.g., levels of arousal and avoidance) and family beliefs (6 items; e.g., the ability to make good treatment decisions). In the current study we included only pre-trauma risk factors (Fig. 1); thus, the two scales of parental stress reaction and family beliefs regarding the child’s medical condition were excluded from the analyses. PAT subscales scores were calculated by dividing the number of endorsed high-risk items by the total number of items in each scale. Adjusted subscale scores ranged from 0 to 1, with higher scores indicating higher psychosocial risk in the specific subscale. The PAT questionnaire has shown strong reliability (Cronbach’s α = 0.8) [40] and has been translated into 13 languages, including Hebrew. The PAT has shown excellent convergent validity in a range of pediatric conditions with standardized measures of family functions, child functioning, and sibling functioning. In the current study a moderate Cronbach’s α was achieved for the total PAT index (=0.67).

#### Dependent variable

##### Mother’s post-traumatic stress symptoms

Mothers’ level of PTSS was evaluated using the Posttraumatic Diagnostic Scale (PDS [42];]. The PDS is a widely used, 17-item self-report questionnaire for assessing PTSS based on DSM-IV criteria for PTSD. All 17 items are summed to create a total score, with higher scores indicating greater PTSS severity, and potential scores ranging from 0 to 51. ﻿ ﻿Psychometric evaluation has demonstrated acceptable to excellent internal consistency, good test–retest reliability, and acceptable convergent and concurrent validity [42]. To obtain an indication of whether mother-reported symptoms were related specifically to their child’s medical event, mothers were asked to complete the questions related to their own PTSS with respect to their child’s medical event, rather than the traumatic event “that bothered them the most”, as in the original version. In the present study, internal reliability was high for mother’s self-report (Cronbach’s α = .92). A PTSD risk cutoff score of ≥23 was obtained as recommended by Sheeran and Zimmerman [43].

### Statistical analyses

Pearson correlation coefficients with a significance level of *p* < 0.05 were conducted to examine the relationship between child- and mother-related factors and the severity of PTSS among mothers in the sample. A radar chart was generated to examine differences in the PAT subscales between Arab and Jewish Israeli mothers. According to the chart, each of the five PAT subscales formed an individual axis of the graph, arranged radially around 0 (no problems). The computed score for each subscale is depicted by the marker on the axis (i.e., spoke); lines connecting the data values for each spoke represented Arab and Jewish mothers’ average subscale score. The closer the marker is to the outer edge of the spoke, the higher the problems reported. Independent t-tests were conducted to examine the effect of ethnicity on mothers’ level of PTSS and the effect of ethnicity on each of the family’s pre-trauma risk factors (i.e., PAT subscales). Finally, following the biopsychosocial model, a multivariate linear regression analysis was conducted to predict mothers’ PTSS with regard to child-related factors, family’s pre-trauma risk factors and mother’s ethnicity. All independent variables were checked for multicollinearity using the variance inflation factor (VIF) (VIF > 10 [44];).

Missing data were managed using SPSS (version 22), using multiple imputation [45]. We used Little’s missing completely at random test [46], with χ^2^(7) = 3.29, *p* = .857, indicating that data were missing completely at random.

## Results

According to our first aim, differences between Israeli Arab and Jewish mothers’ PTSS responses were examined. An independent t-test analysis was conducted to examine ethnic effects (Arab vs. Jewish) on mother’s PTSS following a child’s TME. In accordance with our hypothesis, a significant difference by ethnicity was found, with Arab mothers reporting significantly higher levels of PTSS (M = 20.16, SD = 13.33) than Jewish mothers (M = 13.57, SD = 9.32), *t*_*(82.267)*_ *= − 2.78, p = 0.007*.

Further, we divided the mothers in our sample into two groups of PTSD risk, according to [43] cutoff score of clinical (PTSS > 23) and non-clinical (PTSS < 23) symptomatology. In total, 30% of the mothers in our sample reported significant clinical levels of PTSS, indicating that they are at high risk for developing PTSD. Chi-square analysis revealed significant differences in PTSD risk between the two ethnic groups (χ^2^_*(2)*_ = 7.29, *p* = 0.007), with 44% of Arab mothers at risk for PTSD compared with 19% of Jewish mothers.

With regards to additional child-related factors associated with mothers’ PTSS, the mean difference of sex, gender and type of TME was examined. No significant associations were found between child’s age at TME and mothers’ level of PTSS (*r* = 0.165, *p* = 0.13). In addition, no significant differences were found in mothers’ PTSS between the different etiology groups (*F*_(5,93)_ = 0.66, *p* = 0.65). However, a significant difference between mothers of boys and mothers of girls PTSS scores was found (*t*_*(67.895)*_ *= − 2.25, p = 0.011*), with mothers of boys reporting higher levels of PTSS (M = 18.59, SD = 12.59) than mothers of girls (M = 12.60, SD = 8.85).

**Fig. 2:**
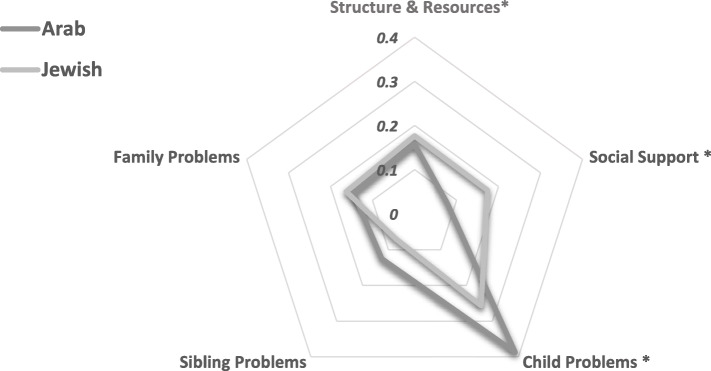
A comparative radar chart representing Arab and Jewish Israeli mothers’ reports on the PAT subscales. Higher scores indicate higher problems reported. * *p* < 0.05

Differences in pre-trauma risk factors between the Arab and Jewish mothers in our sample were examined and are presented in Fig. 2. Additional analysis using independent t-tests revealed a significant difference for the social support subscale, with Arab mothers reporting fewer problems with social support than their Jewish counterparts (*t*_(71.662)_ = 2.21, *p* = 0.03). A significant difference was also observed for the child’s problems factor (*t*_(87.194)_ = − 2.601, *p* = 0.01), with Arab mothers reporting more problems than Jewish mothers. The mean difference between Arab and Jewish mothers in their reports on siblings’ pre-trauma problems approached significance (*t*_(80.529)_ = − 1.84, *p* = 0.07), with Arab mothers reporting more pre-trauma sibling problems than Jewish mothers.

Finally, following the biopsychosocial model, a multivariate regression analysis was applied. In the first step of the model, demographic factors (including child’s biological factors and mothers’ ethnicity) were entered as predictors of mothers’ PTSS. Male gender and Arab ethnicity were positively and significantly related to higher levels of maternal PTSS mother (standardized β-coefficient = 0.224, *p* = 0.02; standardized β-coefficient = 0.268, *p* = 0.07, respectively). The specific model accounted for 15% of the variance of mothers PTSS (*F*_*(3,93)*_ *= 5.285, p = 0.002*). In the second step, pre-trauma risk factors from the PAT questionnaire (i.e., family problems, family structure and resources, child’s and siblings’ emotional and behavioral problems and family’s social support), were included in the regression model as additional predictors. Arab ethnicity and family structure and limited resources were significantly and positively related to mothers’ PTSS (standardized β-coefficient = 0.309, *p* = 0.003; standardized β-coefficient = 0.215, *p* = 0.05, respectively). The total model specified accounted for 25% of the variance in mothers’ PTSS, (*F*_*(8,93)*_ *= 3.558, p = 0.0015*), (see Table 3).

**Table 3:** Multivariate regression model of possible predictors of mothers PTSS

	Regression factor	Unstandardized coefficients ß-coefficients Std. Error		***P***-value
Step 1	Child’s gender (male)	5.869	2.545	0.02
	Age at TME (years)	0.313	0.201	0.12
	Ethnicity (Arab)	6.343	2.305	0.007
	**R = 0.387; model explained variance (R**^**2**^**) = 0.15,** ***F***_***(3,93)***_ ***= 5.285, p = 0.002***			
Step 2	Child’s gender (male)	4.705	2.676	0.08
	Age at TME (years)	0.172	0.204	0.40
	Ethnicity (Arab)	7.332	2.434	0.003
	Structure and resources	17.610	9.023	0.05
	Social support	5.869	5.939	0.32
	Family problems	5.840	6.712	0.39
	Child problems	3.027	5.568	0.59
	Sibling problems	−5.230	9.269	0.58
	**R = 0.501; model explained variance (R**^**2**^**) = 0.25,** ***F***_***(8,93)***_ ***= 3.558, p = 0.001***			

*TME* traumatic medical event

## Discussion

The current study aimed to examine differences in post-traumatic responses between Israeli Arab and Jewish mothers of children following TMEs, as well as the risk and protective factors associated with mothers’ PTSS. Specifically, we used the integrative biosocial framework described by Marsac and colleagues [9] and examined specific child and family risk factors in the context of the two ethnicities.

Our results indicated a significant difference in PTSS between Israeli Arab and Jewish mothers. We found that Arab mothers reported higher post-traumatic symptoms than did their Jewish counterparts. Further, we found that PTSD frequency among Arab mothers (44%) was approximately 2.5 times that of Jewish mothers (19%). These finding are consistent with previous findings indicating cultural differences between Arab and Jewish ethnic groups in their post-traumatic response in multiple settings [28, 36–38]. Similar findings were reported in a study conducted in Sweden, where parents of children with cancer in minority groups (non-Swedish-descendant) showed more stress symptoms than Swedish parents in the sample [47].

The high levels of PTSS reports by the Arab mothers in our study may also be associated with the way illness and disability is perceived within the Arab culture. According to Islam, society is obliged to assess, assist and respect the person with disability and give the person an equal life chance. Mohammad, the Prophet, implied the importance of child welfare, education, well-being, and supporting children other than your own, all which can be seen as the expression of Islamic compassion [48]. However, in a recent review on disability within the Arab world, the authors indicate that regardless of the positive characteristics of those with disabilities, non-disabled individuals within the Arab culture often tend to believe that disabled people are less mature individuals, and that they lack essential skills required for independence. Under this assumption, discrimination, intolerance, and stigma are common attitudes towards individuals with disabilities. The authors further indicate that the negative communication styles and stigma among parents (especially mothers), may impact the methods of education, care and protection for their child with a disability or chronic medical condition [49]. Furthermore, the existence of a previous contact with a person with a disability was a predictive factor in the formation of positive attitudes towards individuals with a disability among young Jewish students, and a negative factor for the Arab participants [50]. Such negative attitudes may increase stress reactions of parents of children following TMEs, and lead to higher levels of PTSS observed in our sub-sample of Arab mothers’.

With regards to gender, mothers of boys in our sample reported higher stress symptoms than did mothers of girls. This finding is in contrast to those of previous reports indicating that following TMEs female gender is a significant risk factor associated with parents’ stress responses [3]. However, it is partially consistent with previous findings in which Arab mothers of boys reported higher post-traumatic symptoms than did mothers of girls [39]. A possible explanation for the male gender difference in mothers’ PTSS found in our study (among both Arab and Jewish mothers) may be related to a more general perspective regarding gender-related values and expectations within Israeli society (both Arab and Jewish) [51]. Rosenthal and Roer-Strier’s [52] ethnocultural study, conducted in Israel, compared Arab and Jewish mothers’ developmental goals for their children and suggested that mothers from both ethnic groups expect their children to demonstrate high levels of self-control, especially regarding negative emotions and behaviors. However, this finding was stronger for mothers’ expectations regarding their daughters, among both Jewish and Arab mothers, than for their expectations regarding their sons. Furthermore, mothers reported having more scholastic and physical-related goals for their sons compared with more sociable and family devotion goals for their daughters [52]. Thus, an injured boy might jeopardize mothers’ future expectations in a more significant way than an injured girl. These differences in gender developmental goals and values may explain the higher levels of symptomatology among mothers of boys in our sample compared with mothers of girls.

Furthermore, we did not find an association between child’s age at trauma and mother’s PTSS. This finding is in contrast to previous studies suggesting that in younger children, the associations between child’s trauma and parental PTSS may be more probable or stronger than in older children [12, 15]. However, most of the studies reporting such associations relate to samples of younger children (0–6 years) (National Center for Child Traumatic Stress, NCTSN.org). It is possible that the fact that most of the children in our sample (70%) were above age 6 contributed to the lack of association between age at TME and mothers’ PTSS.

With regard to injury type or severity, no significant differences were found between the various TME etiologies in our sample and level of maternal PTSS. This finding is consistent with previous findings indicating that parents’ stress symptoms are not predicted by the severity of a child’s illness or injury [19, 53]. This finding is also in line with the pediatric medical traumatic stress model [54], suggesting that the subjective experience of an event has a significantly greater impact on whether it is perceived as traumatic, as compared with any other objective measure of the event, such as injury type or severity.

Among the various pre-trauma risk factors examined, social support and child’s problems were perceived differently by the Arab and Jewish mothers in our sample. Arab mothers perceived having more social support than Jewish mothers did. According to the PAT social support scale, sufficient social support is perceived by individuals if they have someone who can provide child-care, emotional support, financial support and/or information. Empirical research has shown that South Asian families from Muslim backgrounds may use fewer additional support services for their severely impaired children compared to other non-Muslim families [55]. Additionally, previous research indicated that despite widespread governmental support for families with young children in Israel (social security allowance, discounts in kindergartens, etc.), Arab families tend to rely on the support of extended family members and friends and to use fewer external resources when it comes to caring for their children [56]. Duvdevany and Abboud [57] found that the mental well-being of Arab mothers of children with intellectual disabilities in Israel was better when these mothers used more informal support (family and friends) compared with mothers who relied on formal support (i.e., governmental services). This may also be related to the way illness, disability and symptoms are perceived within the Israeli-Arab minority group [26]. Furthermore, Schwartz, Duvdevany and Azaiza [58] argued that Arab mothers tend to first seek assistance in their extended family, under the belief that such assistance will be provided unconditionally. In contrast, Jewish mothers are more likely to turn to a friend, spouse, superior, or professional help providers. Thus, it might be that in our sample, Arab mothers who experienced psychological distress were also more likely to rely on family support [59] and thus perceived their ability to receive social support as unaffected by the child’s TME. However, due to the fact that the proportion of Arab children hospitalized following injuries with severe consequences is higher than that of the Jewish children, [35], the reliance on family members and the use of informal support may not be sufficient. These findings contribute to the limited knowledge available today on the psychosocial differences between Israeli Jews and Arabs as well as on culture-dependent aspects of social support. Our results suggest that social support can be both a protective and a risk factor for PTSS; thus, an in-depth examination of ethnic difference in perceived social support and their effect on mothers of children following TMEs is needed.

Arab mothers in our sample also reported significantly higher levels of child-related problems and approaching-significant levels of sibling-related problems. This finding is in contrast to previous work indicating no significant differences between the reports of Israeli Arab and Jewish parents on a child’s emotional and behavioural problems [60, 61]. Further, compared with Arab caregivers, Jewish caregivers tend to be more open in sharing their feelings about difficulties in caring for their disabled child as well as the impact on other family members [59]. However, several studies showed that following a child’s TME, higher levels of parental stress symptoms are associated with higher reports of the child’s symptoms, suggesting that they might be more prone to overestimating those symptoms [62, 63]. Thus, it is possible that the high levels of child and sibling problems reported by Arab mothers in our sample were protracted by their own relatively high levels of symptomatology [21, 54]. Further studies should aim at collecting information regarding pre-trauma-related factors from additional sources (fathers, health/social care providers, etc.).

Finally, based on the integrative biopsychosocial model [9, 10], the results of our regression model indicated that Arab ethnicity was the strongest predictor of maternal PTSS, and that child’s male gender and a lack of family resources added to the prediction of PTSS among mothers of children following TMEs. Surprisingly, although Arab mothers in our sample perceived higher social support than the Jewish mothers did, it did not serve as a protective factor in our model against the emotional distress associated with their child’s TME. It is possible that while Arab mothers may possess a heightened perceived social support, in specific situations, such as trauma-related events and while being hospitalized away from their social networks, such support does not reduce levels of PTSS [37]. Furthermore, among minority groups, prolonged hospitalization apart from close family networks may increase the pressure and limit the individual’s willingness to obtain help from external sources, prevent support seeking behaviors and inhibit available social resources [27], that can also be provided by the department’s staff [8].

Furthermore, the relation between ethnicity and PTSS in our study warrants some deliberation. First, according to the Israel National Mental Health Survey, Arab-Israelis report higher emotional distress, as compared to their Jewish counterparts (controlling for age and level of education) [64]. The authors claim that the compounded social stress experienced by the Arab-Israeli minority was a contributing factor to their level of emotional distress. Specifically, the Arab population in Israel, is under the pressure of westernization and the need to succeed in a developed country [64]. It is assumed that in this dual process, Arab women may even be more affected than Arab men due to their subordinate status in the traditional, patriarchal society [64]. Furthermore, the vulnerability of Arab–Israeli mothers in our sample to PTSS (above and beyond the Jewish –Israeli mothers) might be related to the breakdown of their sense of social identity in the face of trauma [65]. According to the Social Identity Model of Identity Change (SIMIC) [66], if trauma strengthens a person’s sense of belonging to a valued group, it is more likely it will reduce PTSS risk. However, if the trauma compromises belonging to a valued or meaningful group, or if it contributes to social disconnection, it will tend to increase risk. Furthermore, it is only when a group is relevant to the trauma that social identification with that group mediates the impact of the traumatic experience [66]. In particular, several studies have shown that support has a much greater effect on stress reduction when it is given by someone from one’s own social identity group rather than someone outside of it [67, 68]. Thus, in certain cases, such as that of the Arab mothers in our sample, it may be that although they had perceived their social support as higher than that of the Jewish moms, it did not act as a protective factor against their vulnerability to stress. ﻿.

When these results are considered, the integrative biopsychosocial model indicates that families are best served by medical teams applying an integrative model to understanding, preventing, and treating medical traumatic stress. As suggested by Marsac and colleagues [10], a biopsychosocial approach provides a fuller picture of the development of PTSS, affected by multiple interactions between the various levels of the social-ecological system [69].

Our findings highlight the necessity to include culture and ethnicity in future research regarding parental risk for PTSS following a child’s injury. As indicated in previous studies on parents’ adaptation to their child’s injury among ethnic minority groups [3], our results indicate that it is vital to put greater emphasis on the needs and experiences of Arab parents following their child’s TME when screening for PTSS and PTSD risk. Furthermore, our findings regarding the positive association between family structure and resources and the severity of mothers’ post-traumatic symptoms are also consistent with previous studies in which factors such as parents’ employment status and economic difficulties [3, 70], education level [47, 71] and general family functioning [16] are important predictors of parental PTSS. Thus, adapting the traditional psychological interventions to the values and needs of the Arab minority population in Israel is urgently required.

### Limitations

Several limitations to this study need to be recognized. First, the study is based on mothers’ self-report questionnaires, which can be subject to response biases or self-enhancement [72]. However, for the purposes of this study, self-report was used in order to encourage participants to contribute given that this method is less time-consuming (compared, for example, with the PTSD interview procedure [73]), an important factor in research protocols in the medical setting.

Second, the questionnaires used to assess PTSD in the current study were based on DSM-IV criteria and not on the more recent DSM-V criteria. Because the protocol used to evaluate long-term PTSS was developed in the Pediatric Rehabilitation Department almost a decade ago, it does not include the updated DSM-V version [74]. Although there is a strong correspondence between DSM-IV and DSM-V criteria, the DSM-V is more conservative, and a slight decline in PTSD prevalence might be evident when using those criteria [75]. To keep the questionnaires short and simple for mothers, the research did not use a separate questionnaire to assess social support and only used the PAT social support subscale. This limited our ability to identify different kinds of social support and their effects on mothers’ PTSS; thus, further investigation is needed.

Third, the PAT questionnaire used in the study was translated to Hebrew with the approval of the authors and did not have an Arabic version. Thus, our sample of Israeli-Arab mothers was limited to those who had a proficient Hebrew level. Nonetheless, it should be acknowledged that our sample of Arab mothers with no adequate Hebrew proficiency was approximately 10% of the total sample, reducing the possible bias in our findings.

Finally, in this research we did not fully examine the differences between individuals’ perceived identity, ethnicity and minority status or the religious aspects of each ethnic group. Using a questionnaire to identify participants’ self-perception as a member of a minority or ethnic group can help shed further light on our results and might help identify the risk factor as being linked to participants’ ethnicity. This can help practitioners develop an intervention program based on this important factor.

## Conclusions

Taken together, the current study contributes to the understanding that mental health policies should offer individuals from different ethnic backgrounds the opportunity to approach health services that are sensitive to their cultural values and needs [29]. Values such as interdependence, individuality and use of formal and informal support can differ between minority group members and professionals. Because the effectiveness of professionals working with diverse minority groups is influenced by the culture-specific values and attitudes, a major task for professionals is to understand the wide range of resources, needs and emotional responses of the children and their families. Furthermore, in accordance with the SIMIC, the ability to focus on the shared identities of minority mothers coping with their child’s TME with other individuals or groups (i,e., non-minority mothers) may reduce the negative health-related consequences of the trauma [68, 76]. Such social-oriented “ethnic matching” may contribute to the suitable consumption of health and mental health services by patients belonging to different minority groups [77].

To the best of our knowledge, no study to date has addressed the effect of pre-trauma factors on PTSS among mothers of children following TMEs, in the context of culture and ethnicity. Focusing on such environmental factors within the integrative biopsychological model may help improve our understanding of the mental health needs of mothers from different minority groups and aid in developing appropriate health services and targeted interventions for this population.

## Data Availability

The datasets during and/or analyzed during the current study is available from the corresponding author and all requests for study data and materials will be considered contingent on the Sheba Medical Center Institutional terms.
